# Effects of Nicotine on SH-SY5Y Cells: An NMR-Based Metabolomic Study

**DOI:** 10.3390/metabo15110752

**Published:** 2025-11-20

**Authors:** Enza Napolitano, Carmen Marino, Manuela Grimaldi, Michela Buonocore, Anna Maria D’Ursi

**Affiliations:** 1 Department of Pharmacy, University of Salerno, Via Giovanni Paolo II, 132, 84084 Fisciano, SA, Italy; enapolitano@unisa.it (E.N.); cmarino@unisa.it (C.M.); magrimaldi@unisa.it (M.G.); 2 Department of Chemical Sciences, University of Naples “Federico II” Complesso Universitario Monte Sant’Angelo, Via Cinthia, 80126 Naples, NA, Italy; michela.buonocore@unina.it

**Keywords:** nicotine, cognitive function, SH-SY5Y, Nuclear Magnetic Resonance, metabolomics

## Abstract

**Background/Objectives**: Nicotine is a naturally occurring alkaloid primarily found in *Nicotiana tabacum*. This phytochemical is well known for its addictive properties, and its consumption—particularly through tobacco smoking—is strongly associated with an increased risk of malignancies, metabolic dysfunctions, and cardiovascular as well as respiratory diseases. Despite these adverse effects, several studies have also reported beneficial actions of nicotine, including the enhancement of cognitive functions in several neurodegenerative diseases. **Methods**: To better elucidate the multiple effects of nicotine and clarify their underlying mechanisms, we performed an NMR-based metabolomic analysis of SH-SY5Y neuroblastoma cells exposed to nicotine action. **Results**: Our results indicate that nicotine modulates mitochondrial function and membrane turnover, thereby influencing mitochondrial bioenergetics, synaptic plasticity, and connectivity. **Conclusions**: Collectively, these findings may contribute, at least in part, to explaining the neuroprotective effects of nicotine described in preclinical models of neurodegenerative disease.

## 1. Introduction

Nicotine (IUPAC name (S)-3-(1-methyl-2-pyrrolidinyl)pyridine) is a pyridine alkaloid, which represents approximately 95% of the total alkaloids in the leaves of the tobacco plant (*Nicotiana tabacum*). Nicotine is the main psychoactive component of tobacco smoke, and it has been found to play a key role in starting and maintaining dependence. Therefore, it is often blamed for its link to smoking and addiction. It is well-established that smoking cigarettes elevates the risk of various health conditions, including cancers [1,2], atherosclerotic cardiovascular diseases [3], respiratory diseases [4], and diabetes [5].

Nevertheless, several studies have shown that nicotine has beneficial effects in certain diseases, due to mechanisms other than those underlying addiction [6,7,8]. Interestingly, in Alzheimer’s disease (AD), Parkinson’s disease (PD), age-related memory disorders, schizophrenia, autism, and attention deficit hyperactivity disorder (ADHD), nicotine enhances cognitive functions, including attention, learning, and memory [9,10,11,12].

It is well known that nicotine binds to the nicotinic acetylcholine receptors (nAChRs), and stimulation of different receptor subtypes may lead to different biological effects—i.e., addiction or improvement of cognitive functions [8,9]. Moreover, it has been demonstrated that several distinct biological effects—changes in mitochondrial respiration, cell signaling, and modulation of the inflammatory cascade—may be promoted by nicotine through mechanisms distinct from receptor stimulation [13].

In this context, a diverse set of studies has been carried out to shed light on the multiple effects exerted by nicotine and to provide clarity regarding their origin. Among these, metabolomics analyses have been conducted to evaluate the effects of nicotine in various biological systems, such as the mouse brain and THP-1 monocytes. All these studies pointed to a plethora of effects exerted by nicotine, possibly deriving from receptor stimulation or an alternative biochemical mechanism [14,15]. Metabolomics is an omics science that provides a comprehensive and systematic analysis of multiple metabolite concentrations and their variations in response to external stimuli or genetic mutations. Nuclear Magnetic Resonance (NMR), together with Mass Spectrometry (MS), is the most used technique for studying the metabolomic profiles [16]. In its untargeted approach, metabolomics represents a valuable and effective method for identifying new biomarkers or drug targets, exploring mechanisms of action, and detecting potential off-target effects of drugs. Moreover, metabolomics is finding increasing application in the pharmaceutical field as a novel approach for repurposing existing drugs [17,18,19,20,21,22].

In more detail, the study by Li et al. aimed to investigate the rewarding effects induced by nicotine through the analysis of metabolite extracts from various brain subregions in nicotine-exposed mice. Using NMR-based metabolomic analysis, the researchers found that nicotine significantly altered the brain’s metabolic fingerprint. These changes included disturbances in neurotransmitter levels, alterations in oxidative stress, mitochondrial dysfunction, membrane disruption, imbalances in energy metabolism, and disorders in amino acid profiles [15]. Furthermore, a recent metabolomic study employing mass spectrometry imaging on mouse brain tissue confirmed that chronic nicotine treatment exerts a substantial impact on amino acid pathways and lipid concentrations [23]. In addition, the metabolomic effects of nicotine exposure resulting from tobacco smoke have also been explored in the human brain. While these studies provide valuable insights into nicotine-associated metabolic changes, they do not allow for a clear distinction between the effects of nicotine itself and those of other chemical constituents present in tobacco smoke [24].

To overcome these limitations and extend previous investigations, we chose to examine the effects of nicotine on the human-derived SH-SY5Y cell line, thereby avoiding confounding factors and ethical constraints associated with in vivo models. Compared to the previously studied mouse brain, SH-SY5Y cells—originating from human neuroblastoma—offer genetic stability, reproducibility, scalability, and enhanced translational relevance, as they express functional nicotinic acetylcholine receptors [25,26,27,28,29]. Based on these considerations, our study aimed to characterize the metabolomic profile of SH-SY5Y cells in response to nicotine treatment using NMR spectroscopy. Our data show that nicotine significantly modulates mitochondrial function, particularly affecting the electron transport chain, fatty acid oxidation, and the citric acid cycle. Additionally, nicotine affects membrane turnover, as evidenced by alterations in phospholipid and sphingolipid biosynthesis. Together, these findings suggest a role for nicotine in modulating mitochondrial bioenergetics and, consequently, in processes such as synaptic plasticity and connectivity. Such mechanisms may contribute, at least in part, to the neuroprotective effects of nicotine reported in preclinical models of neurodegenerative disease.

## 2. Materials and Methods

### 2.1. Chemicals

Dulbecco’s Modified Eagle’s Medium (DMEM), L-glutamine, penicillin and streptomycin, fetal bovine serum (FBS), CCK-8, and (-)-nicotine (≥99%) were purchased from Sigma-Aldrich (St. Louis, MI, USA).

### 2.2. Cell Culture

The human neuroblastoma cell line SH-SY5Y was obtained from the American Type Culture Collection (ATCC, Rockville, MD, USA). Cells were cultured in Dulbecco’s Modified Eagle Medium (4500 mg/mL glucose) supplemented with 10% (*v*/*v*) FBS, 2 mM L-glutamine, 100 U/mL penicillin, and 0.1 mg/mL streptomycin. Cells were maintained in a humidified incubator at 37 °C with 5% CO_2_ and were passaged every 2 days.

### 2.3. Cell Viability Assay

Cell viability was assessed by measuring mitochondrial metabolic activity using the Cell Counting Kit-8 (CCK-8, Cat. CK04, Dojindo Laboratories, Rockville, MD, USA) [30], following the manufacturer’s instructions. Briefly, SH-SY5Y cells (8 × 10^3^ per well) were seeded into 96-well plates and incubated for 24 h. Next, nicotine (0.10–10 mM) was added and incubated for a further 24 h. The CCK-8 reagent was then diluted in cell medium (10%) and incubated for 1 h. Absorbance was measured at 450 nm with a microplate reader (Multiskan Go, Thermo Scientific, Waltham, MA, USA).

Cell viability was calculated as the percentage of viable cells relative to untreated control. Data are presented as mean ± standard deviation (SD) from three independent experiments. Statistical analysis was performed using one-way ANOVA followed by Dunnett’s multiple comparisons test, using GraphPad Prism version 8.0 (GraphPad Software, San Diego, CA, USA). Statistical significance was set at *p* < 0.05. Asterisks indicate significance levels compared to untreated cells (CTRL): *p* < 0.01 (**), and *p* < 0.0001 (****). IC_50_ was calculated using GraphPad Prism 8.0 software by nonlinear regression of dose–response inhibition.

### 2.4. ^1^H NMR Metabolomics

#### 2.4.1. Exposure of SH-SY5Y Cells to Nicotine

To prepare metabolomic samples, cells were plated in 60 mm culture dishes and allowed to adhere overnight. Then, 100 µM nicotine was added for 24 h. For the control group, cells were treated only with vehicle (water) for the same duration. All experimental conditions were tested using four biological replicates, each comprising three technical replicates.

#### 2.4.2. Sample Collection and Intracellular Metabolite Extraction

After treatments, both culture media and cell pellet were collected for the metabolomics analysis targeting the exometabolome and endometabolome, respectively. Specifically, the medium was transferred to microcentrifuge tubes and centrifuged at 1000× *g* for 10 min. The same procedure was applied to cell-free medium incubated under identical conditions. The resulting supernatants were transferred to fresh microcentrifuge tubes and stored at −80 °C until NMR analysis. After media removal, cell dishes were washed with cold PBS (pH 7.4) to remove media residues and cells were collected by scraping in methanol. To extract intracellular metabolites from cell pellet, homogenization was followed by biphasic extraction method using methanol, chloroform, and water in a 1:1:1 ratio [31]. Samples were centrifuged at 6000 rpm for 10 min at 4 °C to separate the polar and apolar phases. Polar extracts from cell pellet were dried under vacuum with a SP-Genevac EZ-2 4.0 concentrator (ATS Corporation, Cambridge, ON, Canada) while lipophilic extracts were dried with nitrogen flow for later analysis. All extracts were stored at −80 °C before NMR testing.

#### 2.4.3. NMR Sample Preparation

Lyophilized cell extracts were reconstituted in 200 μL of buffer (50 mM Na_2_HPO_4_, 1 mM trimethylsilyl propionic-2,2,3,3-d_4_ acid sodium salt (TSP-d_4_), 10% of D_2_O). TSP-d_4_ was used as an internal standard for the alignment and quantification of NMR signals. For growth media analysis, 100 μL of cell medium was mixed with 100 μL of the same buffer used for the lyophilized extracts. The resulting samples were transferred into 3 mm NMR tubes for ^1^H NMR acquisition.

#### 2.4.4. NMR Data Acquisition and Processing

1D ^1^H NMR spectra were recorded on a Bruker Ascend™ 600 MHz spectrometer (Bruker Co, Karlsruhi, Germany) equipped with a 5 mm triple resonance Z gradient TXI probe (Bruker Co, Karlsruhi, Germany) at 298 K. One-dimensional NOESY NMR spectra were recorded with 20 k points, 12 ppm spectral width, 1.36s acquisition time, 5 s relaxation delay, 10 ms of mixing time and 128 scans [32]. Topspin version 3.0 (Bruker Biospin, Fällanden, Switzeland) was used for spectrometer control and data processing. Spectra analysis employed an untargeted metabolomic approach, where each metabolite was identified prior to statistical testing using Chenomx NMR-Suite v10.1 (Chenomx NMR Suite, v10.1, Edmonton, AB, Canada). Quantitative analysis of the 1D-NMR spectra was performed using NMRProcFlow ver. 1.4.10 (French National Research Institute for Agriculture, Food and Environment, Bordeaux, France) [33], and the resulting data matrix was subsequently analyzed using statistical tools.

#### 2.4.5. Statistical Analysis

Sample data were normalized using the sum, then log-transformed and Pareto scaled, and analyzed with the MixOmics R package (mixOmics package, ver 6.28.0, University of Melbourne Australia, Melbourne, Victoria, Australia) and Metaboanalyst 6.0 (University of Alberta, Edmonton, Alberta (AB), Canada) [33,34]. Univariate analysis was performed separately on the exometabolome and endometabolome of the groups, using T-test and Fold Change, and results were displayed in a Volcano plot [35].

To enhance the accuracy of the data and gain biological insights, multivariate statistical analysis (MVA) was first applied to the exometabolome and endometabolome concentration matrices, followed by an analysis of the combined datasets. To ensure greater precision in the combined analysis and mitigate the influence of inherent variability between the matrices, the batch effect was eliminated using the R limma package implemented [36]. MVA was conducted on combined matrices of endometabolites and exometabolites using the supervised sparse Partial Least Squares (sPLS), also known as the projection to latent space method [37]. This method is a linear and multivariate visualization technique for integrable datasets that addresses limitations of Principal Component Analysis and Canonical Correspondence analysis (CCA) [38]. In this integrated approach, sPLS analysis is effective when the total number of variables in the combined matrices exceeds the number of samples analyzed, as demonstrated in this study. The sPLS was conducted with a LASSO penalty on the loading vectors to decrease the number of original variables involved in constructing latent variables [39]. A sample plot illustrates the clustering of samples’ metabolomic profiles. In the graph, each sample appears as a point located based on its projection onto the selected latent components of the data. Leave-one-out cross-validation is conducted to validate the model, using R2, Q2, and accuracy metrics [40]. Furthermore, sPLS models were additionally validated using distance matrices derived from the centroid method, maximum distance, and Mahalanobis distance [41].

Variable correlations are displayed using a circular correlation plot, where all vectors are plotted inside a unit circle with a radius of 1. Each vector’s position reflects its correlation with the components; stronger associations produce vectors that extend further from the center. Additionally, variables with vectors close to each other are highly correlated [33]. The contribution of each variable is shown in a bar graph. The contribution graph based on the loadings for variable separation has been color-coded, considering the maximum value between two, thus indicating the clusters where the metabolite has the highest concentration. To comprehensively depict the quantitative changes in metabolites, we created heatmaps using normalized data, average group concentrations, and Euclidean distance [42]. The enrichment pathway tool was used to conduct pathway analysis with Metaboanalyst 6.0. KEGG pathways were selected based on lower false discovery rates (FDR), with *p*-values less than 0.05, and a hit value (the number of metabolites in the pathway) greater than 1 [34].

## 3. Results

### 3.1. Impact of Nicotine on SH-SY5Y Viability

**Figure 1:**
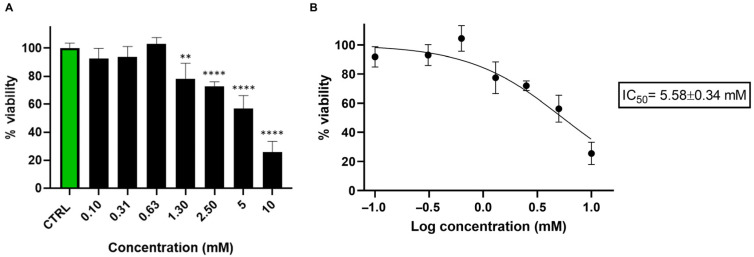
(**A**) Bar graph showing the percentage of viable SH-SY5Y cells 24 h after treatment with nicotine (0.10–10 mM). Cell viability was assessed using the CCK-8 assay and expressed as the percentage of viable cells in treated cultures relative to untreated controls (CTRL). Data are presented as mean ± standard deviation (SD) from three independent experiments. Statistical analysis was performed using one-way ANOVA followed by Dunnett’s multiple comparisons test, using GraphPad Prism 8.0 software (San Diego, CA, USA). Statistical significance was set *p* < 0.05. Asterisks indicate significance levels compared to CTRL: *p* < 0.01 (**), and *p* < 0.0001 (****). (**B**) Nicotine IC_50_ was calculated using GraphPad Prism 8.0 software by nonlinear regression of dose–response inhibition.

Initially, we performed a viability test to determine the effect of nicotine on SH-SY5Y cell survival. Since metabolomic analysis results depend heavily on treatment conditions, we deemed this preliminary step essential. The viability test helps us identify the optimal nicotine concentration and exposure duration for sample preparation in metabolomics analysis. The bar graph reported in Figure 1A indicates that nicotine significantly impacts cell viability at concentrations above 1.30 mM, with an IC_50_ of 5.58 ± 0.34 mM (Figure 1B).

### 3.2. Nicotine Influences Lipid Metabolism, Mitochondria Function, and Amino Acid Concentrations

^1^H-NMR spectroscopy was employed to investigate the metabolomic profiles of cellular extracts and growth medium, representing the endometabolome and exometabolome, respectively. SH-SY5Y cells were treated with 100 µM nicotine for 24 h prior to sample collection for metabolomic analysis. This concentration was selected based on cell viability assays, which confirmed that it does not compromise SH-SY5Y cell viability. It was therefore considered appropriate for investigating potential metabolomic alterations induced by nicotine under non-cytotoxic conditions.

**Figure 2:**
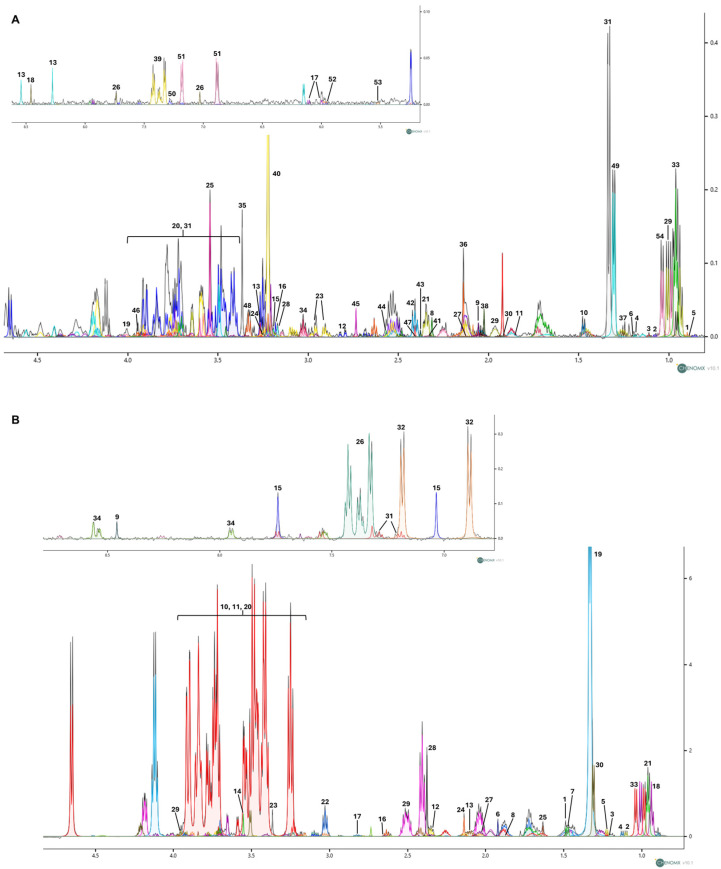
(**A**) Representative 1D ^1^H NOESY spectrum illustrating the polar cellular extracts (endometabolome) obtained from SH-SY5Y cells treated with nicotine. A total of 54 metabolites were identified in the endometabolome: 1: 2-Hydroxybutyric acid; 2: 2-Methyl-3-ketovaleric acid; 3: 2-Oxobutyrate; 4: 3-Hydroxybutyrate; 5: 3-Methyl-2-oxovalerate; 6: 5,6-Dihydrothymine; 7: Acetate; 8: Acetoacetate; 9: Acetylcysteine; 10: Alanine; 11: Arginine; 12: Aspartate; 13: ATP; 14: Betaine; 15: Carnitine; 16: Choline; 17: Citicoline; 18: Formate; 19: Fructose; 20: Glucose; 21: Glutamate; 22: Glutamine; 23: Glutathione; 24: Glycerophosphocholine; 25: Glycine; 26: Histidine; 27: Homocysteine; 28: Isobutyryl-L-carnitine; 29: Isoleucine; 30: Isovalerate; 31: Lactate; 32: Lactose; 33: Leucine; 34: Lysine; 35: Methanol; 36: Methionine; 37: Methylmalonate; 38: N-Acetyl-L-aspartic acid; 39: Phenylalanine; 40: Phosphorylcholine; 41: Proline; 42: Pyroglutamate; 43: Pyruvate; 44: Riboflavin; 45: Sarcosine; 46: Serine; 47: Succinate; 48: Taurine; 49: Threonine; 50: Tryptophan; 51: Tyrosine; 52: UDP-glucose; 53: UDP-N-Acetylglucosamine; 54: Valine. (**B**) Representative 1D ^1^H NOESY spectrum of growth medium (exometabolome) obtained from SH-SY5Y cells treated with nicotine. A total of 34 metabolites were identified in the exometabolome: 1: 2-Aminoisobutyric acid; 2: 2-Methyl-3-ketovaleric acid; 3: 3-Hydroxybutyrate; 4: 3-Methyl-2-oxovalerate; 5: 5,6-Dihydrothymine; 6: Acetate; 7: Alanine; 8: Arginine; 9: Formate; 10: Fructose; 11: Glucose; 12: Glutamate; 13:Glutamine; 14: Glycine; 15: Histidine; 16: Homocysteine; 17: Homocystine; 18: Isoleucine; 19: Lactate; 20: Lactose; 21: Leucine; 22: Lysine; 23: Methanol; 24: Methionine; 25: N-Methyl-a-aminoisobutyric acid; 26: Phenylalanine; 27: Proline; 28: Pyroglutamate; 29: Serine; 30: Threonine; 31: Tryptophan; 32: Tyrosine; 33: Valine; 34: Nicotine. Spectra were acquired at 600 MHz. The different colours denote individual metabolites identified with the Chenomx NMR Suite v10.1 software.

Figure 2A,B show the representative 1D ^1^H NOESY NMR spectra [31] of cellular endometabolome and exometabolome, respectively. Through ^1^H chemical shift assignment, a total of 54 metabolites were identified in the endometabolome and 34 in the exometabolome.

After normalization by sum, Log transformation, and Pareto scaling, the concentration data matrices were analyzed using both univariate and multivariate approaches.

**Figure 3:**
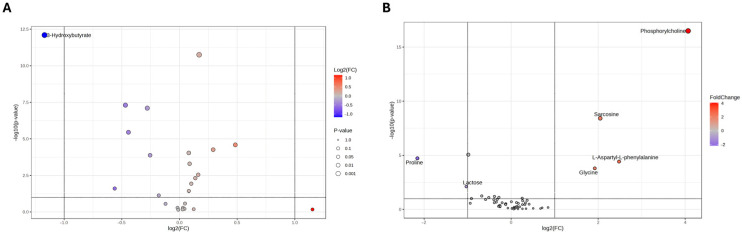
Volcano plot analysis of metabolic changes in the exometabolome (**A**) and endometabolome (**B**) of SH-SY5Y cells treated with nicotine vs. untreated cells (CTRL). Each point on the volcano plot was based on *p*-value and fold-change value, set at 0.05 and 2.0, respectively. Red points identify upregulated metabolites, whereas blue points identify down-regulated metabolites.

**Figure 4:**
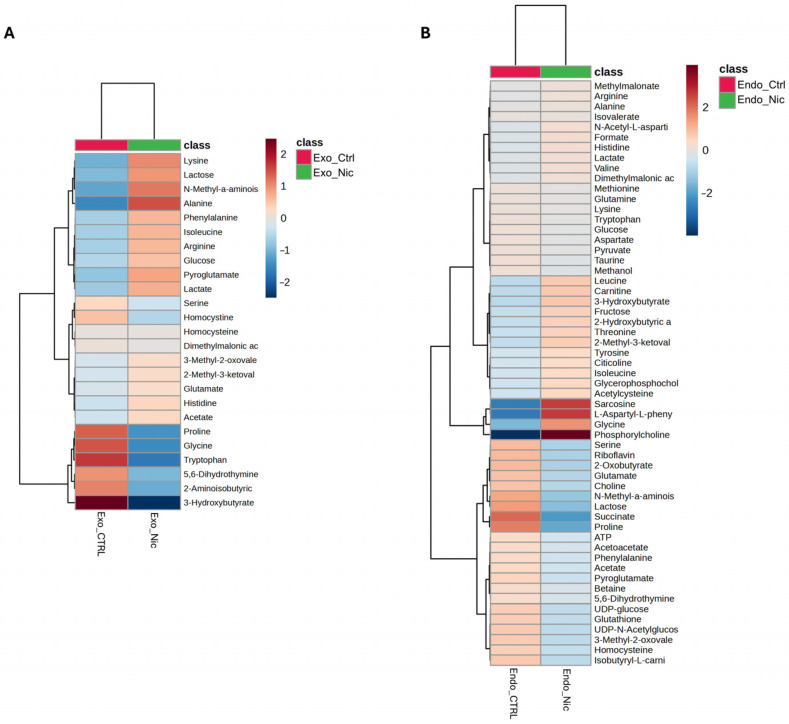
Heatmaps of changed metabolites in the exometabolome (**A**) and (**B**) endometabolome. The color of each section corresponds to a concentration value of each metabolite calculated by a normalized concentration matrix (red, up-regulated; blue, down-regulated).

As shown in the Volcano plot in Figure 3A, the extracellular medium of SH-SY5Y cells treated with nicotine exhibits a lower concentration of 3-hydroxybutyrate. On the other hand, the intracellular compartment (Figure 3B) reports higher concentrations of glycine, L-aspartyl-phenylalanine, sarcosine, and phosphorylcholine (PC), while lower concentrations of lactose and proline. A comprehensive overview of the relative concentrations of all metabolites is in the heatmaps presented in Figure 4A,B.

The metabolic equilibrium between the intracellular and extracellular compartments is crucial in assessing the biological function of a biological system. Accordingly, we performed a combined metabolomic analysis of endometabolome and exometabolome, providing a more accurate understanding of the cells’ metabolic behavior after nicotine treatment.

**Figure 5:**
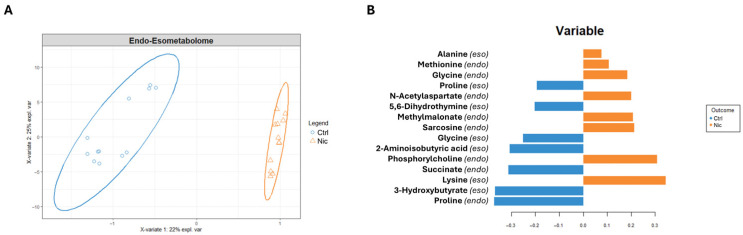
(**A**) sPLS-DA score scatter plots related to the metabolomic profile of combined cellular compartments of SH-SY5Y treated with nicotine for 24 h (Nic) vs. control cells (Ctrl). The cluster analyses are reported in the Cartesian space described by the principal components PC1:22% and PC2:25%. sPLS-DA was evaluated using cross-validation (CV) analysis. CV tests performed according to the sPLS-DA statistical protocol show a significant cluster separation (0.98 and 0.79 accuracy values on PC1 and PC2, with positive 0.58 and 0.55 Q2 indices, respectively). (**B**) Loadings bar plot related to the combined matrices of endometabolome and exometabolome. The variables responsible for metabolomic profile differences are ordered according to values of increasing importance from bottom to top. Colors indicate the cluster where the median is maximum for each metabolite (orange: nicotine; blue: Control).

Therefore, after an initial exploration of the separate cell compartments, we conducted a supervised, integrated analysis using the Sparse Partial Least Squares Determination Analysis (sPLS-DA) approach. Figure 5A shows a sPLS-DA score plot representing the metabolomic profile of the combined cellular compartments of SH-SY5Y cells treated with nicotine and control cells exposed to the vehicle. The Cartesian space, described by the first and second principal components (PC1 and PC2), explains 29% and 22% of the dataset variance, consistent with a net metabolomic difference between the endometabolomes and exometabolomes of treated and untreated cells. The model’s validity was evaluated using a cross-validation approach, based on Q2 parameter (0.58 and 0.55 Q2 indices on PC1 and PC2, respectively). Furthermore, the separation model area was validated by the calculation of the Mahalanobis distance, maximum distance, and centroids (Figure S1). In addition, ROC analysis yielded an area under the curve (AUC) of 100%, confirming the robustness of the model for both component 1 and component 2 (Figure S2).

The bar plot (Figure 5B), reporting the discriminating metabolites classified by their loading values, indicates that nicotine-exposed cells are characterized in the extracellular environment by a higher concentration of alanine and lysine, and a lower concentration of proline, 5,6-dihydrothymine, glycine, 2-aminoisobutyric acid, and 3-hydroxybutyrate. Conversely, in response to nicotine treatment, the intracellular compartment shows increased concentrations of methionine, glycine, N-acetylaspartate (NAA), sarcosine, PC, and reduced concentrations of succinate and proline.

**Figure 6:**
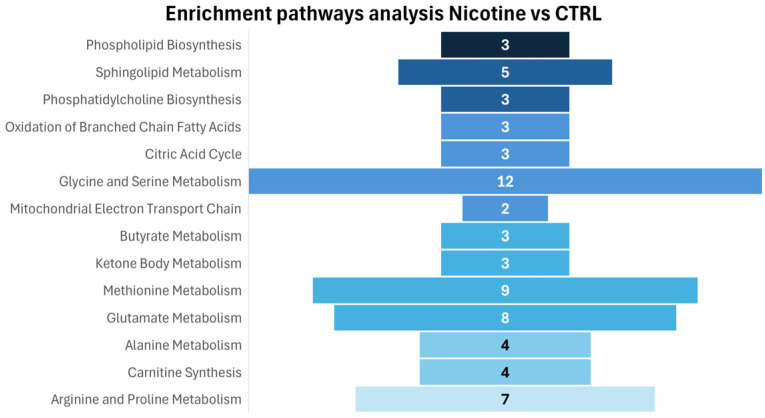
Enrichment pathways analysis: the discriminative pathways are ranked according to *p*-value and number of hits reported in the bars.

Figure 6 presents the pathway enrichment analysis performed on intracellular metabolites to identify the biochemical pathways affected by nicotine treatment, in accordance with the observed metabolite alterations.

Consistent with a significant increase in phosphorylcholine concentration, enrichment analysis reveals an effect on biochemical pathways related to phospholipid biosynthesis, specifically sphingolipid biosynthesis and phosphatidylcholine biosynthesis, highlighting a role of nicotine in inducing important lipid remodeling, modifying cell lipid metabolism, and cell membrane structure. On the other hand, evident effects are observable in amino acid metabolism (methionine, arginine, and proline metabolism; glycine and serine metabolism; glutamate and alanine metabolism) and mitochondrial activity, particularly in the mitochondrial electron transport chain, oxidation of branched-chain fatty acids, and citric acid metabolism.

## 4. Discussion

Nicotine, the primary alkaloid in *Nicotiana tabacum*, is considered the main psychoactive component of tobacco smoke. It significantly contributes to dependence and addiction. However, it is widely accepted that nicotine enhances brain function and cognitive performance. Some research suggests that this effect is partly due to nicotine’s influence on mitochondrial activity, although the complete mechanism has not been fully elucidated [43,44,45,46].

Metabolomics studies aimed at investigating the impact of nicotine on mice’s brains have been previously carried out [15,23]. In the present work, we have reported an NMR-based pharmacometabolomic study on an in vitro system represented by the SH-SY5Y cell line. These human neuroblastoma cells are widely used in neuroscience experiments and are frequently employed for studying nicotine-related mechanisms, as they express nicotinic acetylcholine receptors (nAChRs) [25,26,27,28,29].

Our data show a significant impact of nicotine on lipid metabolism and cellular membrane architecture. In particular, exposure of SH-SY5Y cell cultures to nicotine led to a marked elevation of intracellular concentrations of PC, which is a critical precursor in the biosynthesis of phosphatidylcholine. Given the predominant role of phosphatidylcholine in animal cell membranes, its precursor, PC, represents a robust biomarker of phospholipid synthesis—a process essential for neural membrane assembly and dendritic extension during brain development. Consistent with this role, previous studies employing ^31^P NMR spectroscopy have demonstrated a pronounced increase in PC levels throughout neuronal maturation, indicative of enhanced membrane biogenesis and remodeling. These dynamic processes are fundamental for the establishment of synaptic connectivity and the initiation of neuronal electrical activity [47]. This interpretation is further supported by recent findings showing that increased availability of phospholipid precursors promotes neuronal membrane expansion and synaptogenesis [48].

The ability of nicotine to modulate phospholipid metabolism aligns with recent lipidomic studies demonstrating its neuroprotective effects. For instance, in SH-SY5Y cells exposed to 6-hydroxydopamine (6-OHDA) to model Parkinson’s disease, nicotine was shown to normalize lipid metabolism via α7 nAChR-mediated signaling and anti-inflammatory pathways. These findings underscore nicotine’s capacity to reprogram lipid metabolism under neurodegenerative conditions [49]. Moreover, our observations are consistent with previous in vivo studies examining the metabolomic profile of mouse brain tissues in response to nicotine exposure [15,23]. Despite future validation being needed to confirm this hypothesis, collectively, these data support the notion that upregulation of phospholipid synthesis—facilitating membrane remodeling—is likely to enhance synaptic connectivity and neural plasticity, mechanisms that may underlie the cognitive-enhancing effects attributed to nicotine.

In accordance with data previously collected in preclinical studies [15], the treatment of SH-SY5Y cells with nicotine affects several biochemical pathways related to amino acid metabolism, specifically those involving methionine, arginine, proline, glycine, serine, glutamate, and alanine. The interpretation of this effect is challenging, and several hypotheses have been proposed regarding whether the alterations in amino acid concentration could be related to changes in energetic metabolism and/or synaptic neurotransmission.

Notably, the observed increase in glycine and sarcosine following nicotine administration may reflect neuroprotective mechanisms. Glycine plays a crucial role in modulating excitatory neurotransmission, as it functions as a co-agonist of NMDA receptors [50,51]. This effect appears to be further supported by the concomitant rise in sarcosine, which contributes to elevating glycine levels and potentiating NMDA receptor activity—a process essential for learning, memory, and synaptic plasticity [41,42]. In addition, sarcosine has been shown to exert neuroprotective effects by attenuating glutamate-induced toxicity in SH-SY5Y cells, thereby supporting its potential role in mitigating excitotoxic damage [52].

Several pieces of evidence have demonstrated nicotine’s ability to influence mitochondrial activity [46]. It has been proposed that nicotine may exert its effects by binding to nicotinic acetylcholine receptors (nAChRs) located on the outer mitochondrial membrane (OMM), whose presence has been identified on isolated mitochondria from mouse liver. These receptors may serve as binding sites for nicotine and contribute to the modulation of mitochondrial signaling and function [53].

Independently of the precise site of action, our findings confirm that nicotine treatment modulates multiple biochemical pathways associated with mitochondrial function.

Among these, the most significantly affected are oxidation of branched-chain fatty acids (*p*-value: 1.83 × 10^−5^), citric acid metabolism (*p*-value: 6.47 × 10^−5^), mitochondrial electron transport chain (*p*-value: 8.79 × 10^−5^) stand out as the most significantly affected pathways. Moreover, nicotine administration was associated with a reduction in intracellular succinate levels. Previous studies in rodents have demonstrated that both acute and chronic nicotine exposure elicit marked hyperactivities of mitochondrial dehydrogenases, specifically malate dehydrogenase (MDH) and succinate dehydrogenase (SDH), within the brain [54]. The observed decrease in intracellular succinate in human cells may therefore reflect a comparable nicotine-induced enhancement of SDH activity.

Being the biological space where ATP is produced through oxidative phosphorylation (OXPHOS), mitochondria are organelles with a pivotal role in human cells, often referred to as the cell’s powerhouses. Studies conducted by Cormier et al. on mitochondria isolated from rat forebrain revealed that nicotine binds to complex I and inhibits its NADH-ubiquinone reductase activity, as demonstrated by in vitro oxygen consumption binding assays. This interaction impairs the electron flow from NADH to complex I, resulting in reduced mitochondrial oxygen consumption [55]. Additionally, Wang et al. reported that in the rat brain, chronic nicotine administration modulates the expression of several genes encoding subunits of protein complexes involved in the mitochondrial respiratory chain [56]. Our findings confirm that nicotine can also modulate the electron transport chain in human neuronal-like cells, supporting the notion that mitochondria are a key target of nicotine’s action. However, further investigation is required to clarify the underlying mechanisms of nicotine’s effects on human mitochondrial function.

Additional evidence for nicotine’s action at the mitochondrial level is provided by the observed increase in N-acetylaspartate (NAA) concentration. NAA is widely regarded as a biomarker of neuronal integrity, with reduced levels typically reflecting neuronal loss or impaired neuronal function [57]. In our study, NAA was found to increase in response to nicotine treatment, a change that may indicate improved neuronal viability and mitochondrial activity. Consistent with this observation, elevated NAA concentrations have been reported in the brains of smokers, suggesting that nicotine exposure may contribute, at least in part, to this effect [58].

Collectively, these findings indicate that nicotine modulates key mitochondrial processes related to energy metabolism and cellular function in neuronal-like cells, consistent with observations from both in vivo animal models and human studies.

While our findings provide valuable insights into the effects of nicotine on neuron-like cells, several limitations must be acknowledged. SH-SY5Y cells, derived from human neuroblastoma, exhibit an immature neuronal phenotype, which may not fully recapitulate the responses of mature neurons in vivo [59]. Additionally, their use in monoculture fails to capture the complex interactions with glial cells, which are critical for neuronal function and homeostasis [60]. Moreover, this study was conducted under non-pathological conditions, limiting its applicability to disease models where neuronal metabolism is altered.

Despite these limitations, SH-SY5Y cells remain a useful tool for early-stage mechanistic studies. Notably, their low expression of the α4β2 nicotinic receptor subtype enables a more targeted investigation of alternative receptors, such as α7, which has been implicated in neuroprotective pathways. Future studies should aim to validate these findings in more physiologically relevant systems, including differentiated neurons, co-culture models, or in vivo approaches, particularly in the context of neurodegenerative diseases like Alzheimer’s and Parkinson’s.

## 5. Conclusions

**Figure 7:**
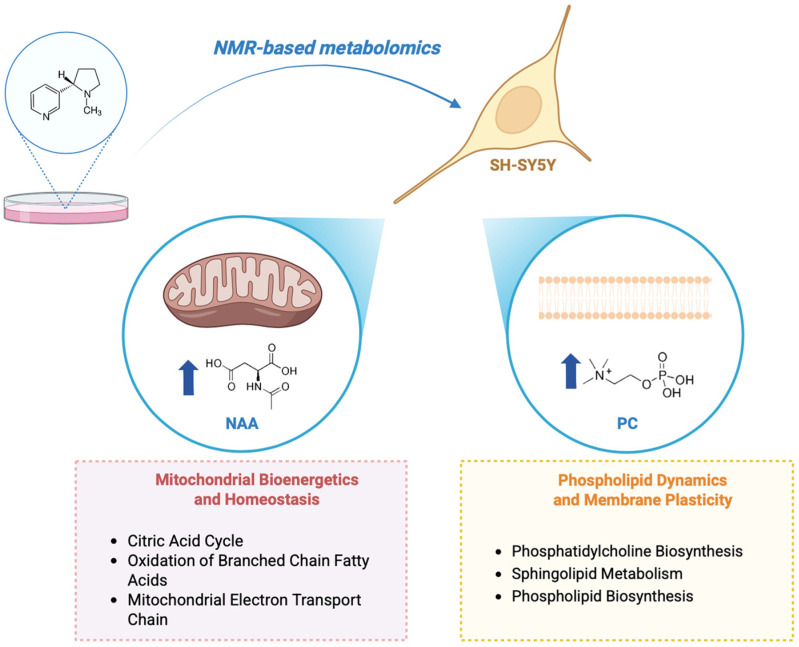
Schematic representation of nicotine-induced effects on SH-SY5Y neuron-like cells, illustrating key metabolic and functional changes. The upward arrows show a rise in concentration. This figure was created using BioRender, https://www.biorender.com/.

In conclusion, our results demonstrate that nicotine significantly alters the metabolic profile of SH-SY5Y neuron-like cells through two main mechanisms, as illustrated in Figure 7:

(i) Regulation of membrane dynamics and plasticity. Nicotine treatment increases intracellular phosphorylcholine (PC) and modulates pathways related to phospholipid and sphingolipid metabolism. These alterations suggest enhanced membrane remodeling and synaptic plasticity, potentially contributing to nicotine’s cognitive effects.
(ii) Modulation of mitochondrial bioenergetics and homeostasis. Nicotine influences key mitochondrial pathways and elevates N-acetylaspartate (NAA) levels, supporting improved mitochondrial activity and neuronal viability. These findings align with previous evidence and highlight mitochondria as a central target of nicotine’s cellular mechanism of action.

The identification of specific metabolic pathways affected by nicotine opens potential avenues for therapeutic intervention in neurodegenerative disorders or cognitive impairments, where mitochondrial dysfunction and altered membrane dynamics are commonly implicated.

## Data Availability

Metabolomics data have been deposited to the EMBL-EBI MetaboLights database (https://www.ebi.ac.uk/metabolights/, accessed on 11 August 2025) with the identifier MTBLS12848 [61].

## Supplementary Materials

The following supporting information can be downloaded at: https://www.mdpi.com/article/10.3390/metabo15110752/s1, Figure S1. Sample prediction area plot created using Maximum distance, Centroid and Mahalanobis showing the distribution of samples in validation areas related to Nicotine-24H vs. CTRL (a–c). Figure S2. The validation of the separation model was performed using combined endometabolome and exometabolome matrices of nicotine-treated and untreated cells. The following data include only the area values under the curve and the validation results based on *p*-values. Table S1. Pathway Enrichment analysis discriminates between the analyzed clusters. The number of hits corresponds to the number of metabolites detected in the spectrum that participate in the biochemical pathways and are explicit in the column ‘metabolites’. Raw p represents the significance validation index reporting the *p*-value; Holm Bonferroni represents the adjustment of the *p*-value for the number of analyzed samples (Holm p.); the FDR index calculates the number of False Discovery Rates. Biochemical pathways with hits > 2 and Raw p, Holm p, FDR < 0.05 were considered significant.
