# Supplementary material for: Effects of Nicotine on SH-SY5Y Cells: An NMR-Based Metabolomic Study
Source: Metabolites. 2025 Nov 20;15(11):752. doi: 10.3390/metabo15110752 (PMC12654241; doi:10.3390/metabo15110752)
Supplement: Supplementary file 1 [file metabolites-15-00752-s001.zip › metabolites-3949531-supplementary.pdf]

Effects of nicotine on SH-SY5Y cells: an NMR-based metabolomic study

Enza Napolitano [1,2], Carmen Marino [1], Manuela Grimaldi [1], Michela Buonocore [3] and Anna Maria D'Ursi [1,*]

1 Department of Pharmacy, University of Salerno, Via Giovanni Paolo II, 132, 84084 Fisciano, Salerno, Italy; enapolitano@unisa.it; cmarino@unisa.it; magrimaldi@unisa.it.

2 PhD Program in Drug Discovery and Development, University of Salerno, Via Giovanni Paolo II, 132, 84084, Fisciano, Salerno, Italy.

3 Department of Chemical Sciences, University of Naples "Federico II", Complesso Universitario Monte Sant'Angelo, Via Cinthia, Naples, 80126, Italy; michela.buonocore@unina.it

* Correspondence: dursi@unisa.it.

Table of contents

Figure S1. Sample prediction area plot created using Maximum distance, Centroid and Mahalanobis showing the distribution of samples in validation areas related to Nicotine-24H vs CTRL (**a,b,c**).

Figure S2. The validation of the separation model was performed using combined endo- and exo-metabolome matrices of nicotine-treated and untreated cells. The following data include only the area values under the curve and the validation results based on p-values.

Table S1. Pathway Enrichment analysis discriminates between the analysed clusters. The number of hits corresponds to the number of metabolites detected in the spectrum that participate in the biochemical pathways and are explicit in the column ‘metabolites’. Raw p represents the significance validation index reporting the p-value; Holm Bonferroni represents the adjustment of the p.value for the number of analysed samples (Holm p.); the FDR index calculates the number of False Discovery Rates. Biochemical pathways with hits>2 and Raw.p, Holm p, FDR <0.05 were considered significant.

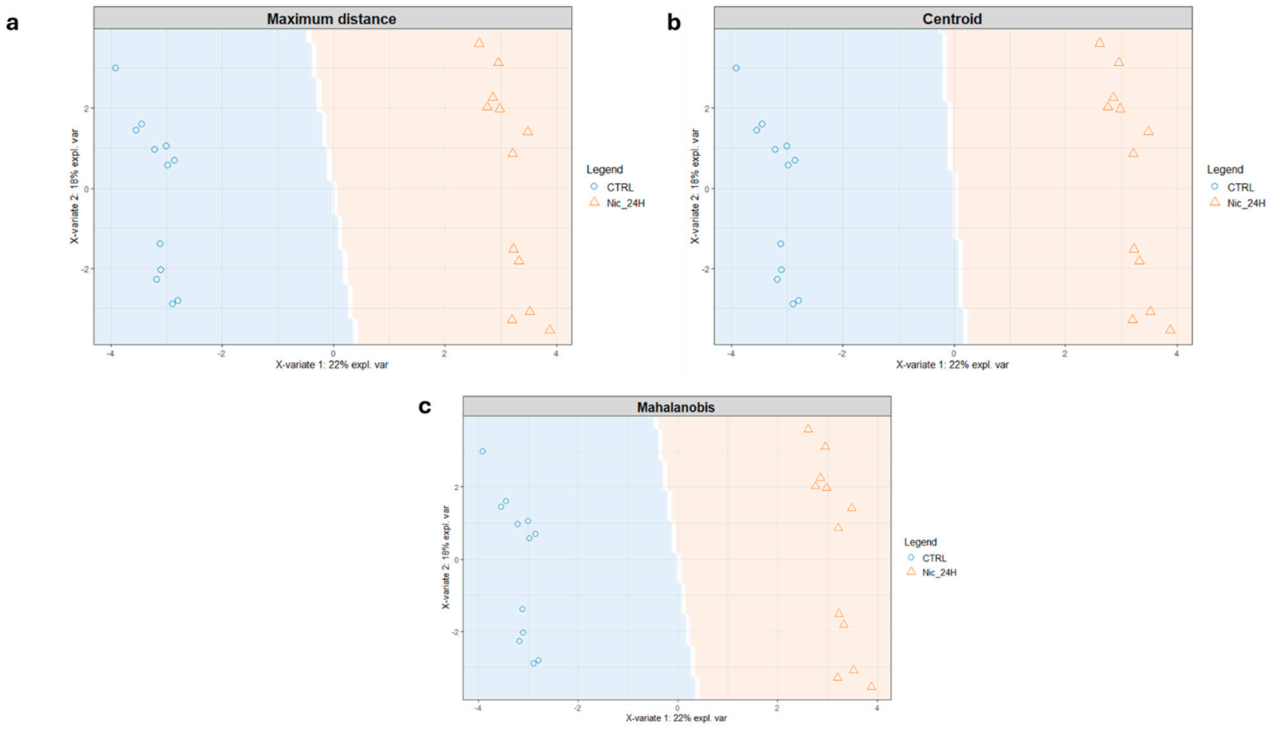

Figure S1. Sample prediction area plot created using Maximum distance, Centroid and Mahalanobis showing the distribution of samples in validation areas related to Nicotine-24H vs CTRL (**a,b,c**).

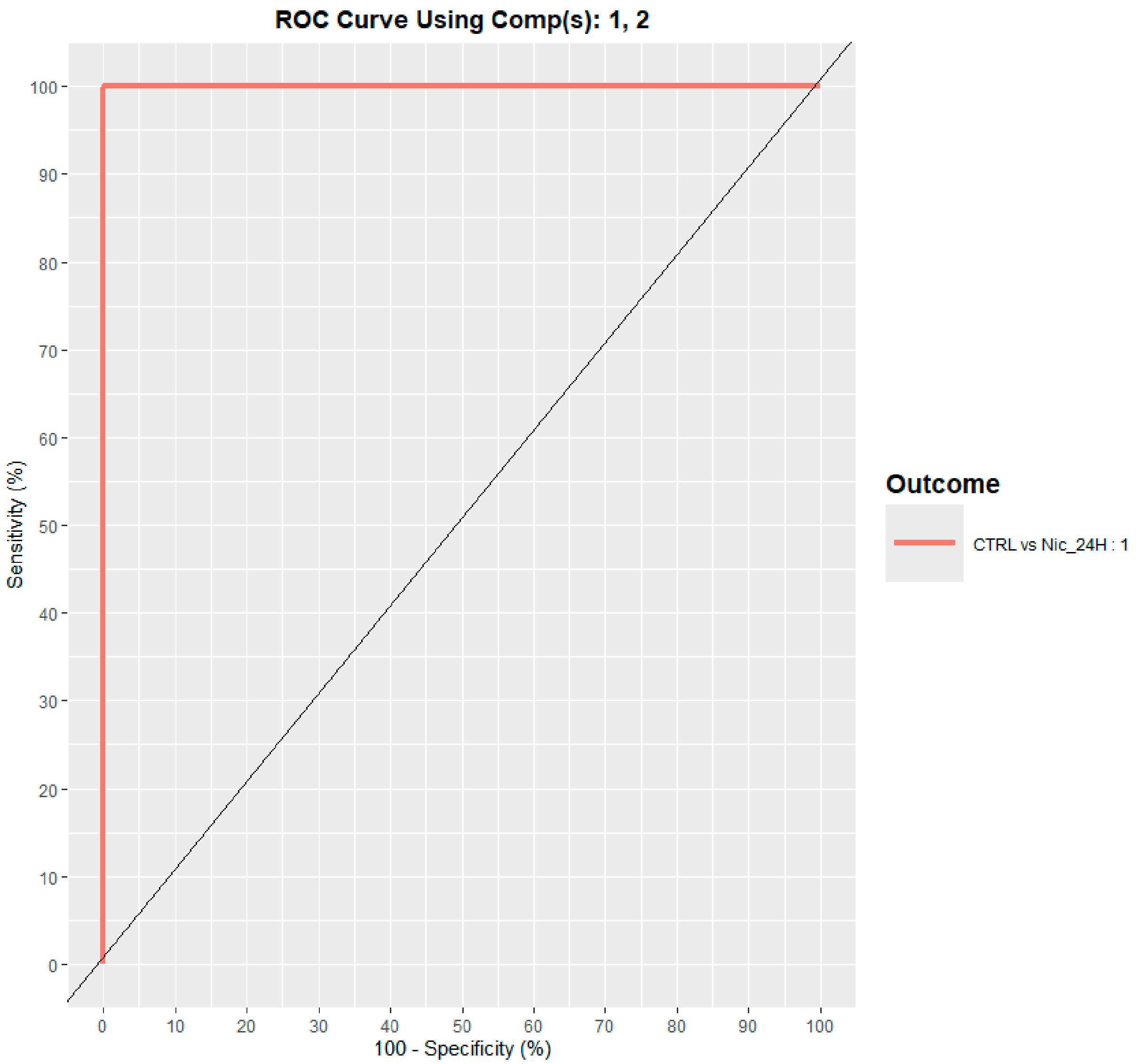

Component	*AUC*	*p.value*
**Comp 1**	1	3.26e-05
**Comp 2**	1	3.26e-05

Figure S2. The validation of the separation model was performed using combined endo- and exo-metabolome matrices of nicotine-treated and untreated cells. The following data include only the area values under the curve and the validation results based on p-values.

Table S1. Pathway Enrichment analysis discriminates between the analysed clusters. The number of hits corresponds to the number of metabolites detected in the spectrum that participate in the biochemical pathways and are explicit in the column ‘metabolites’. Raw p represents the significance validation index reporting the p-value; Holm Bonferroni represents the adjustment of the p.value for the number of analysed samples (Holm p.); the FDR index calculates the number of False Discovery Rates. Biochemical pathways with hits>2 and Raw.p, Holm p, FDR <0.05 were considered significant.

	Hits	Raw p	Holm p	FDR	
Phospholipid Biosynthesis	3	1,31E-11	1,12E-09	1,12E-09	Phosphorilcholine, Choline,Citilcoline
Sphingolipid Metabolism	5	3,26E-10	2,77E-08	1,36E-08	Uridine diphosphate glucose,Phoshorilcholine,Serine,Glucose,ATP
Phosphatidylcholine Biosynthesis	3	4,75E-10	3,99E-08	1,36E-08	Phosphorilcholine, Choline,ATP
Oxidation of Branched Chain Fatty Acids	3	1,83E-05	0.0014267	0.00017477	Succinic acid; Adenosine triphosphate;Carnitine
Citric Acid Cycle	3	6,47E-05	0.0049849	0.00050665	Pyruvic acid; Succinic acid; Adenosine triphosphate
Glycine and Serine Metabolism	12	6,48E-05	0.0049849	0.00050665	2-Ketobutyric acid; Betaine;Glycine;Glutamic acid;L-Threonine; Serine;Pyruvic acid;Sarcosine;L-Arginine; Adenosine triphosphate;Methionine; Homocysteine;
Mitochondrial Electron Transport Chain	2	8,79E-05	0.00070282	9,44E-03	Succinic acid; Adenosine triphosphate;
Butyrate Metabolism	3	3,58E-04	0.026458	0.0023652	Acetoacetic acid; Succinic acid; Adenosine triphosphate
Ketone Body Metabolism	3	5,68E-04	0.041449	0.0034879	3-Hydroxybutyric acid; Acetoacetic acid; Succinic acid
Methionine Metabolism	9	7,64E-04	6,34E-03	1,64E-02	2-Ketobutyric acid; Betaine;Glycine,Serine,Sarcosine,ATP,Methionine,Homocysteine,Choline
Glutamate Metabolism	8	8,34E-04	0.060073	0.0047836	Glutathione; Glutamic acid; Glycine;L-Aspartic acid; Pyruvic acid;Succinic acid;Adenosine triphosphate;Glutamine
Alanine Metabolism	4	4,61E-03	0.32736	0.024016	Glycine; Glutamic acid Pyruvic acid; Adenosine triphosphate
Carnitine Synthesis	4	4,69E-03	3,85E-01	8,07E-02	L-Carnitine; Glycine; Lysine; Succinic acid
Arginine and Proline Metabolism	7	4,52E-02	0.0003662	6,48E-01	Glutamic acid; Proline; L-Aspartic acid; Succinic acidL-Arginine; Adenosine triphosphate;Glycine
